# Trends and Challenges in Tumor Anti-Angiogenic Therapies

**DOI:** 10.3390/cells8091102

**Published:** 2019-09-18

**Authors:** József Jászai, Mirko H.H. Schmidt

**Affiliations:** 1 Institute of Anatomy, Medical Faculty Carl Gustav Carus, Technische Universität Dresden School of Medicine, 01307 Dresden, Germany; mhhs@mailbox.tu-dresden.de; 2 German Cancer Consortium (DKTK), Partner Site Dresden, 01307 Dresden, Germany; 3 German Cancer Research Center (DKFZ), 61920 Heidelberg, Germany

**Keywords:** anti-angiogenesis therapy of cancer, sprouting angiogenesis, stromal microenviroment, evasive resistance, vessel normalization, anti-VEGF therapy, Bevacizumab, Aflibercept, small-molecule multikinase-inhibitors, angiogenesis inhibitors

## Abstract

Excessive abnormal angiogenesis plays a pivotal role in tumor progression and is a hallmark of solid tumors. This process is driven by an imbalance between pro- and anti-angiogenic factors dominated by the tissue hypoxia-triggered overproduction of vascular endothelial growth factor (VEGF). VEGF-mediated signaling has quickly become one of the most promising anti-angiogenic therapeutic targets in oncology. Nevertheless, the clinical efficacy of this approach is severely limited in certain tumor types or shows only transient efficacy in patients. Acquired or intrinsic therapy resistance associated with anti-VEGF monotherapeutic approaches indicates the necessity of a paradigm change when targeting neoangiogenesis in solid tumors. In this context, the elaboration of the conceptual framework of “vessel normalization” might be a promising approach to increase the efficacy of anti-angiogenic therapies and the survival rates of patients. Indeed, the promotion of vessel maturation instead of regressing tumors by vaso-obliteration could result in reduced tumor hypoxia and improved drug delivery. The implementation of such anti-angiogenic strategies, however, faces several pitfalls due to the potential involvement of multiple pro-angiogenic factors and modulatory effects of the innate and adaptive immune system. Thus, effective treatments bypassing relapses associated with anti-VEGF monotherapies or breaking the intrinsic therapy resistance of solid tumors might use combination therapies or agents with a multimodal mode of action. This review enumerates some of the current approaches and possible future directions of treating solid tumors by targeting neovascularization.

## 1. Introduction

Tumorigenesis is a multistep process in which genetic and epigenetic mechanisms lead to the dysregulation of proto-oncogenes and tumor suppressor genes initiating the malignant transformation of cells [1]. Dictated by the increasing metabolic demand and tissue hypoxia, neoplasms require neoangiogenesis for their progressive growth and metastasis, irrespective of the initial genetic lesion or environmental insult causing the malignant transformation [2,3,4,5,6]. Postulates of Judah Folkman concerning tumor angiogenesis as a potential therapeutic target shifted the emphasis from traditional tumor cell-centered therapeutic strategies towards anti-angiogenic approaches, establishing a new field in oncology [2,7,8,9,10,11]. Milestone discoveries were made concerning the identification of angiogenic factors, the regulation of neoangiogenesis and the development of anti-angiogenic therapeutic modalities that could interfere with pathological angiogenesis. Although a number of pro-angiogenic factors were identified, VEGF was established as the key mediator of pathological angiogenesis in several scenarios [12,13]. Not surprisingly, targeting the VEGF/VEGFR signaling axis has become central to the development of anti-angiogenic medicine. Information from over 3000 registered clinical trials can be retrieved with the key words “tumor anti-angiogenic” from the ClinicalTrials.gov database run at the National Institutes of Health, and about 2000 hits are found with the key word combination “anti-VEGF tumor”. Numerous anti-angiogenic drugs with disparate molecule structures have been developed and gained regulatory approval for cancer treatment [14,15,16,17,18] and for that of ocular neovascular diseases sharing molecular pathways with tumor angiogenesis [15,19]. Therapies for cancer focusing exclusively on inhibiting new vessel growth and/or destroying pre-existing vessels remain, however, suboptimal or have shown limited clinical efficacy [20,21,22]. Moreover, the inhibition of tumor angiogenesis, for instance, could paradoxically lead to the selective survival of hypoxic cancer cells, especially in the center of the tumor mass. In addition, the ablation of a given angiogenic factor or a particular inflammatory cell type might evoke compensatory reactions by eliciting the compensatory secretion of alternative angiogenic factors [23,24,25,26,27,28,29] or by the attraction of another cell type with a pro-inflammatory/pro-angiogenic phenotype [30]. Thus, the adaptive resistance/compensatory refractoriness might severely limit the success of single-target monotherapeutic approaches. Due to the high proportion of non-responder patients with solid tumors with intrinsic or acquired resistance in conjunction with anti-VEGF treatments, there is an unmet need for novel strategies to compensate for the shortcomings of current therapeutic modalities [15]. The present review addresses topics of neovascularization, relevant factors of pathological angiogenesis, and possible cellular/molecular confounder factors underlying the limited efficacy of current anti-angiogenic approaches and discusses some novel avenues to overcome resistance.

## 2. Mechanisms of Angiogenesis

### 2.1. “Angiogenic Switch”

**Figure 1:**
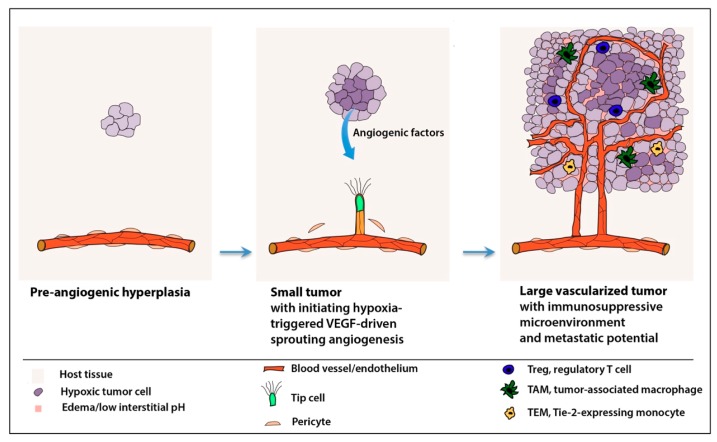
Steps of tissue hypoxia-triggered neoangiogenesis and progression of neoplastic lesions. VEGF: vascular endothelial growth factor. The initially avascular tumor cell mass, upon reaching a critical size, cannot ensure its further growth without the trophic support of its own circulation. Hypoxic cells of the tumor mass, via angiogenic factors, initiate the “angiogenic switch” by stimulating nearby endothelial cells of the microvasculature of the host/parental tissue in which they arise. Tumor angiogenesis initiates with a detachment of perivascular cells, the degradation of vessel basal lamina and angiogenic sprouting by the formation of filopodia bearing leading-edge endothelial tip cells from the parental vessels. Newly formed sprouts build anastomoses followed by lumen formation and the recruitment of the pericytic cells necessary for perivascular investment. Angiogenesis will continue as the expansive growth of the tumor mass requires further supply. This process forms a vicious circle that is fueled by the tumor vessel leakiness and the suboptimal tumor perfusion that cannot efficiently relief tissue hypoxia. Recruited inflammatory cells contribute significantly to a hostile microenvironment that boosts further uncontrolled neoangiogenesis and play a role in the evasive resistance of solid tumors. Abbreviations: Treg, regulatory T cell; TAM, tumor-associated macrophage; TEM, Tie-2- expressing monocyte.

In order to keep up with the changes of metabolic demand that the further propagation and growth of the tumor cell mass pose, cells of the neoplasm must acquire their own microcirculation (Figure 1) [2,3,4]. Once the cell congregate (i.e., the hyperplastic cell mass) reaches a critical size, its nutrient and oxygen supply or waste product removal, as a function of the increasing distance from the nearest existing vessels, cannot be covered by blood vessels provided by the natural microenviroment of the tissue in which the population of tumor cells arises. In this process, tumor and endothelial cells within the neoplasm may constitute a “highly integrated ecosystem” depending on each other [2]. In a broader sense, cells composing the tumor stroma (i.e., tumor-associated fibroblasts, perivascular and inflammatory cells) supported by the alteration of the microenvironment elicit the complex multistep process of neoangiogenesis. The new tumor-nourishing microvessels arise from pre-existing ones of the host circulation governed by a net balance of positive and negative regulators of blood vessel growth [2,31,32,33,34]. Although this rate-limiting event of tumorigenesis, often termed as the “angiogenic switch”, seems to be a discrete step, neoangiogenesis accompanies not only the transition phase from avascular hyperplasia to vascularized neoplasia but remains present during the further progression of tumorigenesis to a practically unlimited degree. Progressive neoangiogenesis supports the further expansion and metastasis of tumors [3,4,35]. Detailed reviews on the topic tackle the possible mechanisms by which the neoplastic cells might gain access to the parental/host circulatory system [32,35,36,37].

### 2.2. Normal vs. Pathological Angiogenesis—Similar, yet Distinct

Organized vascularization is essential for organogenesis. During development, the blood circulatory system is among the first organ systems to arise. However, physiologically active (adaptive) angiogenesis in adult tissue is a rare phenomenon. This is mainly restricted to biological processes connected to the female reproductive cycle (endometrium and ovarian folliculogenesis and the post-ovulatory development of the corpus luteum) and tissue repair associated with wound healing and inflammation [6,9,38]. By contrast, the dysregulation of angiogenesis is a significant feature of a number of pathological conditions. Besides tumor neoangiogenesis, deregulated abnormal angiogenesis has been observed in vasoproliferative retinopathies, rheumatoid arthritis, stroke, and myocardial infarction [39,40,41].

It is well known that hypoxia functions as a key trigger of angiogenesis both under physiological and pathological conditions, and the expression of VEGF is chiefly driven by its hypoxia responsiveness [42]. What makes tumor angiogenesis abnormal is a pivotal question. One of the major differences between tumor angiogenesis and physiological angiogenesis is the way that the microvasculature copes with the nourishment of cells in need. Physiological angiogenesis in adulthood, as with developmental angiogenesis, represents a self-limiting process; that is, it is resolved when the microvascular network perfusion becomes (re-)established. This brings the relief of tissue hypoxia [43]. In spite of the fact that tumors share some fundamental molecular mechanisms driving angiogenesis in the physiological context of tissue defense, renewal or repair, neoangiogenesis in solid tumors leads to the formation of abnormal vessel beds [20,44,45]. In solid tumors, tissue hypoxia is further increased as a consequence of suboptimal tumor perfusion by structurally and functionally irregular neovessels, and an indefinite vicious circle is established with a persistent aberrant angiogenic regulatory loop [6,20]. Thus, in contrast to physiological tissue repair processes, in which vasopermeability, inflammatory infiltration and neovessel formation/remodeling are self-limiting, tumor-associated pathological angiogenesis remains unresolved, and tumors behave as if they were “wounds that never heal” [46]. In turn, tumor cells undergo a metabolic reprogramming and adapt to—for normal somatic cells—an unfavorable hypoxic and low pH microenvironment [6,33,47,48] (see also Section 4.2.). Tissue hypoxia represents the major trigger of proangiogenic factor production. Hypoxia fuels the secretion of a number of proangiogenic factors not only by tumor cells, but also by macrophages and stromal fibroblasts [21,48,49,50]. One of the most important down-stream molecular mechanisms regulating the expression of proangiogenic factors, including that of VEGF, is hypoxia-inducible factor-1 (HIF-1), a master regulator of oxygen homeostasis [42,51,52,53,54]. Sprouting angiogenesis is initiated when cells of a given tissue respond to hypoxia and quiescent endothelial cells (henceforth ECs) sense proangiogenic signals (Figure 1) [53,55,56]. Under the influence of angiogenic factors released by hypoxic, inflammatory or tumor cells, the detachment of pericytes occurs via an Angiopoietin-2 (Ang-2) mediated mechanism. This is accompanied by the proteolytic degradation of the basement membrane by matrix-metalloproteinases (MMPs), and the endothelial cell–cell junction will be loosened [55,57,58].

A key event in sprouting angiogenesis is the selection of motile leading-edge tip cells (Figure 1). This process is governed by a VEGF gradient acting on an equivalence group of ECs (i.e., a set of cells with the same potential to adopt a particular activation state), whereby ECs sense VEGF-A through VEGFR-2 and respond by acquiring a tip cell status accompanied by the migration towards hypoxic regions to form new blood vessels [59,60,61]. Tip cells, instead of being a lineage with fixed cell fate, represent a particular functional activation state of endothelial cells. However, this activation state is coupled with some specific morphological features including the presence of numerous actin-based filopodial extensions [59]. Pioneering tip cells, stimulated by VEGF-A gradients, serve as sensors of pro-angiogenic cues as they express receptors of key angiogenic factors (e.g., VEGFR-2). Moreover, they actively participate in degrading the extracellular matrix by secreting the proteolytic enzymes necessary for the execution of the sprouting angiogenesis program [55,59,62]. This process is regulated by the down-modulation of Notch-signaling, which appears to be a direct (patho)physiological antagonist of VEGF action in this particular context [61,63,64,65,66,67]. Delta-like 4 (Dll4), with high expression levels in tip cells, binds Notch, which in turn induces the proteolytic release of the Notch intracellular domain (NICD) in neighboring ECs. This leads to the differential regulation of VEGFR-1 and -2 expression, resulting in reduced VEGF-A sensitivity and the promotion of stalk cell behavior [63]. ECs dynamically compete for the tip cell position during angiogenic sprouting [59]. The orderly, directional expansion of the vascularization front requires the formation of trailing stalk cells for the elongation of the new sprout by proliferation [68]. The adjacent phalanx cells are quiescent and mark the mature region of a blood vessel [68].

The resolution of the angiogenic process is achieved by returning to a non-proliferating phalanx phenotype [55] whereby tip cells lose their “proteolytic phenotype“. ECs and perivascular cells express metalloprotease inhibitors upon the reestablishment of the contact between both cell types [6]. Increased expression of VEGFR-1 reduces the proliferative and migratory capacities of cells, and the enhanced expression of platelet-derived growth factor B (PDGF-B) by ECs facilitates the recruitment of PDGF receptor β (PDGFR-β)-expressing pericytes, which are necessary for the vessel to stabilize perivascular coat formation [55,58]. Angiopoietin-1 (Ang1) expression by perivascular cells further enhances the association of pericytes with stalk cells through a tyrosine kinase receptor (tyrosine kinase with immunoglobulin-like and EGF-like domains), Tie-2 [69]. Vascular sprouts behind the vascularization front fuse, followed by the formation of new, lumenized vessels. Tissue macrophages/microglia contribute to angiogenesis by serving as chaperones in the formation of vascular anastomoses [70]. Thereafter, the maturation process starts and remodeling takes place [55]. Under pathological conditions, however, the process is deregulated, with a significant contribution from the hostile stromal microenvironment and the accessory cellular components.

Although VEGF-A-activated receptor tyrosine kinase signaling is a crucial and often rate-limiting step, both in physiological and tumor angiogenesis, it is not the only pathway in the context of (neo)angiogenesis. Whereas in pathological angiogenesis, including tumor angiogenesis, hypoxia and VEGF are central to the acquisition of the angiogenic switch, alternative mechanisms leading to hypoxia-inducible factor 1-alpha (HIF-1α)-activation without hypoxia might also belong to the repertoire of an angiogenic phenotype in tumors [12,53]. Thus, the loss of tumor-suppressor function of von Hippel Lindau tumor-suppressor protein (pVHL) by stabilizing HIF-1α up-regulates the expression of a number of hypoxia-responsive factors including VEGF-A. This phenomenon is referred to as “hypoxic mimicry” and has been described in patients with clear-cell renal carcinoma (RCC), to name only the most prevalent condition [12,53].

Angiogenesis is a highly regulated multistep process which requires a concerted action of multiple cytokines/growth factors, extracellular matrix and cell adhesion molecules as well as various cell types [55,58,71,72]. In addition to VEGF-A, further members of the VEGF-family and their cognate receptor tyrosine kinases and co-receptors, as well as other growth factors/molecules, were reported to play a role in pathological angiogenesis. Thus, in spite of its dispensable contribution to developmental angiogenesis, the secreted VEGF-family member placental-growth factor (PLGF) was shown to play a modulatory/auxiliary role in neovascularization [73] (see also Section 3.1). Moreover, several alternative factors with angiogenic activity, partly secreted by inflammatory cells, have been identified as affecting neovascularization: acidic fibroblast growth factor (aFGF/FGF-1), basic FGF (bFGF/FGF-2), transforming growth factor-α (TGF-α), TGF-β, hepatocyte growth factor (HGF), tumor necrosis factor-α (TNF-α), monocyte chemotactic protein 1 (MCP-1), angiogenin, interleukin-8 (IL-8), angiopoietins and EGFL7 [13,16,31,55,74,75]. Besides secreted cytokines, enzymes were also described to be significantly involved in neoangiogenesis/tumor growth, including MMP9, which is mainly produced by tumor-infiltrating myeloid cells, and cyclo-oxygenase-2 (Cox-2), which is known for producing eicosanoide inflammatory mediators under pathologic conditions, with little if any contribution to developmental vessel formation [4,76].

The number of activities a given factor might exert adds a further level of complexity to the regulation of the angionic process. Thus, VEGF-A, besides being a potent vasopermeability factor, promotes vascular sprouting, induces the secretion of ECM-degrading enzymes, and protects neovessels from apoptosis [12]. In addition, the degree of angiogenic effects of VEGF-A might be tissue-specific or context-dependent [77]. Furthermore, these effects are pivotally influenced by the bioavailability of VEGF-A, which is fine-tuned by an interaction with ECM and is dependent on a competition with a secreted ECM-binding molecule, Esm-1 [78].

## 3. Angiogenic Factors and Signaling Pathways of Tumor Angiogenesis

### 3.1. VEGF Family and VEGFR Signaling

**Figure 2:**
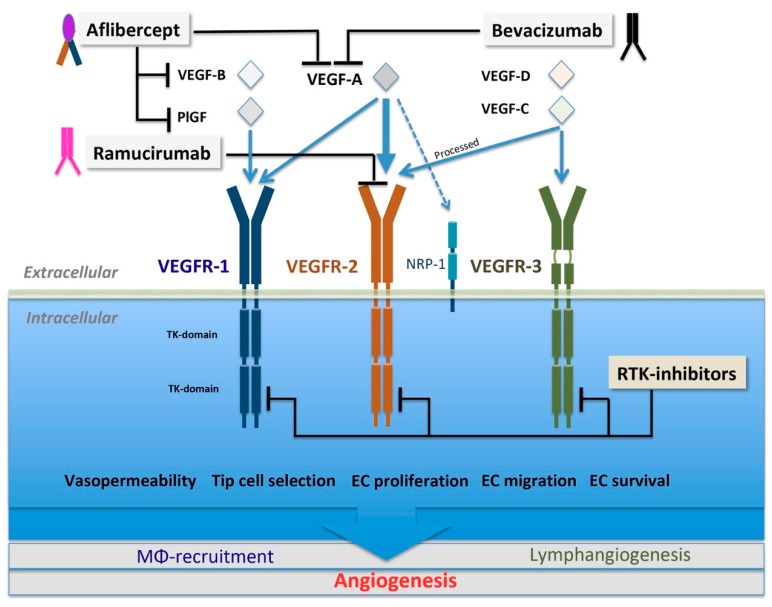
The VEGF/VEGFR signaling axis, its contribution to neoangiogenesis and treatment modalities interfering with its activity. Binding of VEGF ligands to their cognate receptors leads to receptor dimerization and autophosphorylation triggering a down-stream intracellular phosphorylation cascade. In principle, tumor anti-angiogenesis can be achieved (1) by prohibiting ligand binding to their cognate TK receptors (VEGFR1-3) receptors/non-tyrosine kinase (NRPs) co-receptors either by the withdrawal of pro-angiogenic ligands of the VEGF family (Aflibercept, Bevacizumab) or by blocking the accessibility of the binding pocket for ligands on a particular receptor (Ramucirumab); (2) by interfering with the kinase activity of VEGFRs (small molecule multikinase inhibitors: receptor tyrosine kinase (RTK)-inhibitors). Anti-VEGF-targeted therapies result in the inhibition of down-stream signaling mechanisms, governing a range of steps involved in neovessel formation and/or inflammatory infiltration. Abbreviations: EC, endothelial cell; MΦ, macrophage; PlGF, placental growth factor; RTK, receptor tyrosine kinase; TK-domain, tyrosine kinase-domain; VEGF, vascular endothelial growth factor; VEGFR, vascular endothelial growth factor receptor.

The most important and best-characterized pro-angiogenic molecular factors and signaling pathways involved in tumor neoangiogenesis are the members of the VEGF-family, which act through their cognate tyrosine kinase receptors and certain co-receptors (Figure 2). The VEGF family of secreted, dimeric glycoproteins comprises five members in mammals, designated as VEGF-A, B, C, D and PlGF (Figure 2) [15,79]. Structurally and functionally related, further, non-vertebrate members of the VEGF family are encoded in the parapox-virus genome or found in snake venom [79]. Three VEGF receptors have been described in mammals, namely VEGFR-1 (fms-like TK 1, Flt1), VEGFR-2 (kinase insert domain receptor, KDR) and VEGFR-3 (fms-like TK 4, Flt4) (Figure 2), all belonging to class IV of tyrosine kinase (TK) receptors [79]. Besides TK receptors, VEGFs are also known to bind neuropilins as co-receptors. Neuropilins (NRPs) form a small, two-member family of non-tyrosine kinase receptors, termed as NRP-1 and NRP-2. These co-receptor molecules comprise long extracellular regions, single transmembrane domains, and short cytoplasmic sequences [79]. NRPs, besides their co-receptor role for members of the VEGF family, are also known as receptors for class III semaphorins—polypeptides with key roles in neuronal axon guidance [80].

Historically, VEGF was isolated as a potent vasopermeability factor (VPF), and molecular cloning revealed that this molecule is a secreted heparin-binding protein related to PDGF with angiogenic mitogen activity [81,82,83,84]. VPF/VEGF is a multifunctional cytokine, and the tissue hypoxia-triggered over-activation of VEGF expression is not only a common denominator, but also the most critical regulator of a number of neovascular pathologies including tumor angiogenesis [12,42,85]. VEGF, besides its mitogen function and promotion of vascular sprouting in pathological neovascularization, also acts as an important survival factor for ECs and immature vessels in vivo and is necessary for protecting neovessels from apoptosis [12,81,86,87]. Moreover, VEGF induces the secretion of ECM-degrading enzymes (e.g., collagenases, urokinase-type plasminogen activator) [12]. While its ability to render microvascular ECs hyperpermeable, in contrast to other angiogenic mitogens, is extremely high and is several thousand-fold higher than that of histamine, its mitogenic activity lags behind that of other pro-angiogenic factors [81,88]. The plasma leakage elicited by VEGF is an important step in generating a provisory fibrin-matrix for the attraction of inflammatory cells and in supporting the ingrowth of new blood vessels, resulting in the formation of a mature, vascularized stroma [76,89].

In order to distinguish VEGF (i.e., the founder of the VEGF superfamily of growth factors) from other structurally related members of this molecule family, it is often denoted as VEGF-A. However, VEGF-A itself does not represent a single mature molecular species; it is a group of molecules appearing in several alternatively spliced isoforms. Among the VEGF-A isoforms with relevance to both physiological and pathological angiogenesis, splice form VEGF-A165 appears to be the most critical factor in pathological neovascularization [14,90,91,92,93]. VEGF-A isoforms, amounting altogether to 16 to date, differ in their length and are distinguished by the indices standing behind their name, which reflects the length of the particular molecule. It is important to note that alternative splicing is not an exclusive property of VEGF-A; spliced isoforms were also observed for two further VEGF-superfamily members, VEGF-B and PlGF. Alternative splicing has an immense functional relevance given that distinct VEGF-A isoforms differ significantly in their heparin-binding affinity, which has a crucial influence over their bioavailability and interactions with defined co-receptors [94]. Beyond the molecular diversity generated at the transcriptional level, post-translational processes further increase the plurality within the VEGF-family. The reason for this is a fine-tuning/diversification of the biological effects that a particular molecular subtype could exert. Thus, post-translational proteolytic processing, in addition to the molecular diversity generated by alternative RNA splicing, might have a further impact on receptor-binding affinity and interactions with the ECM. This substantially modifies the spreading and biological availability of certain members of this secreted growth factor family [14,15,79,90,95,96]. VEGF-superfamily members can be expressed and secreted by several cell types including tumor cells, tumor-infiltrating inflammatory cells and cancer-associated fibroblasts [55,90]. Although VEGF-A (especially VEGF-A165)-triggered VEGFR-2 signaling is clearly central to the pathogenesis of neovascularization, both in cancer and diabetic retinopathy, it is not the sole pathway involved in neoangiogenesis [73,90,97,98].

Alternative avenues leading to pathological neovascularization either in a direct or indirect manner have been described involving members of the VEGF-superfamily other than VEGF-A. Several lines of evidence suggest that PlGF has been linked to pathological angiogenesis in different organs [73,98,99,100]. While PlGF levels are low or undetectable in healthy tissues, a significant upregulation is observed under pathological conditions [101,102]. In line with this, PlGF is apparently dispensable for vascular development, but it plays a central role in pathological angiogenesis. Consequently, it might represent an interesting alternative therapeutic target in ischemic, malignant and inflammatory conditions [103]. Its main activity is the potentiation of VEGF/VEGFR-2 signaling by preventing the binding of VEGF-A to VEGFR1 and the upregulation of pro-angiogenic factors [73,104,105,106]. The potentiation of VEGFR-2 signaling by PlGF is achieved in an indirect way by outcompeting VEGF-A from VEGFR-1 binding sites, thus redirecting VEGF-A towards VEGFR-2 receptor binding sites, for which PlGF possesses only a very low affinity [107,108,109]. It is worthy of note that the binding-affinity of VEGFR-1 for VEGF-A is also significantly (one order of magnitude) higher than that of VEGFR-2; however, its kinase activity is about 10-fold weaker than that of VEGFR-2 [97]. PlGF, acting through VEGFR-1 expressed on macrophages, regulates inflammatory infiltration in pathological conditions, thus contributing to the pro-angiogenic cytokine microenvironment by promoting the release of additional angiogenic factors necessary for neoangiogenesis [110,111,112]. In this particular context, VEGFR-1 activation is primarily required for the recruitment of hematopoietic precursors and the migration of monocytes and macrophages [14].

A further member of the VEGF-family, VEGF-C, was found to play a role in angiogenesis. In contrast to PlGF, VEGF-C makes a more significant contribution to developmental vessel formation, while its involvement in pathological angiogenesis is also not negligible. Thus, besides its well- known developmental action in lymphangiogenesis via VEGFR-3 signaling [113], VEGF-C contributes to angiogenesis by activating VEGFR-3 or via VEGFR-2 following a proteolytic cleavage [8,114,115]. Under pathological conditions, infiltrating inflammatory cells might represent a major source of VEGF-C [116]. The secretion of VEGF-C was also observed in a hypoxia-independent manner as a compensatory mechanism in response to anti-VEGF therapies [25,29,117]. VEGF-C is capable of inducing vascular permeability, EC proliferation and migration [116,118].

Besides VEGF-C, VEGF-D has been shown to regulate vascular and lymphatic EC function via the activation of VEGFR-3 [119]. Upon proteolytic processing, however, similar to VEGF-C, VEGF-D acquires the capacity to bind and activate VEGFR-2. However, the affinity of VEGFR-2 for these ligands is much lower than that for VEGF-A [96]. Nevertheless, in the context of pathological responses, the potential binding of these VEGF-family members cannot be neglected. This requires treatment strategies that instead of modulating pathological VEGF-A levels by means of the major anti-VEGF-A monoclonal antibody, bevacizumab, block VEGFR-2-mediated pathological responses by inhibiting the access of all possible VEGF-family ligands to this receptor by a novel VEGFR-2 specific antibody tool (Figure 2; ramucirumab).

The function of further VEGF family members, and that of co-receptors, is less clearly defined and not free of controversies. Thus, the angiogenic potential of VEGF-B appears very weak in most tissues, and VEGF-B-deficient mice are viable and display no overt morphological phenotype [95]. Alternative splicing generates two isoforms of VEGF-B [120]. VEGF-B is speculated to act as a pro-survival factor for ECs via NRP 1 and VEGFR-1 [121]. NRPs were found to be highly expressed in tumor cell lines and neoplasms and have been linked to tumor growth and neovascularization [122]. NRP-1 and NRP-2 lack cytoplasmic enzyme activity, but bind VEGFs and semaphorins through two distinct extracellular domains and form complexes with other transmembrane receptors regulating down-stream signal transmission [80].

### 3.2. Alternative Angiogenic Factors

#### 3.2.1. FGF Family and FGF Receptors

**Figure 3:**
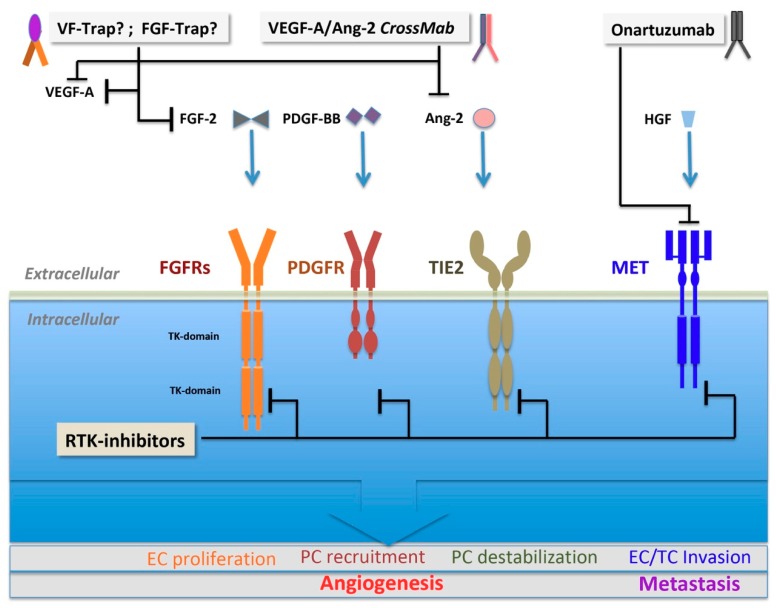
Alternative angiogenic pathways playing a role in tumor angiogenesis, refractoriness, evasive resistance or relapses in response to anti-VEGF monotherapies and tools available for targeting their effects. Resistance to anti-angiogenic therapy might be mediated by angiogenic factors that trigger a switch from an initially VEGF-dependent state to a VEGF-independent angiogenic process. In order to interfere with these alternative pathways, besides the small molecule multikinase inhibitors (RTK-inhibitors) blocking down-stream signal cascade activation even in the presence of receptor occupancy, several recombinant biological tools have been developed. The range of these tools includes decoys (“ligand traps”) aimed at withdrawing alternative pro-angiogenic factors either alone (fibroblast growth factor (FGF)-Trap) or simultaneously with VEGF-A (VF-Trap), engineered multivalent monoclonals (CrossMabs) or antibody tools raised against RTK receptors blocking the access of ligands to their cognate receptor (onartuzumab). Abbreviations: EC, endothelial cell; PC, pericyte; TC, tumor cell; Ang-2, angiopoietin-2; FGF-2, fibroblast growth factor-2; FGFR, fibroblast growth factor receptor; HGF, hepatocyte growth factor; MET, mesenchymal-epithelial transition proto-oncogene; PDGF, platelet-derived growth factor; PDGFR, platelet-derived growth factor receptor; TIE2, Tyrosine kinase with immunoglobulin-like and EGF-like domains 2; RTK, receptor tyrosine kinase.

The mammalian fibroblast growth factor (FGF) family contains 22 genes, 18 of which encode molecules known to perform signaling with FGF tyrosine kinase receptors, FGFR-1–4. Heparin-binding is crucial for the function of these factors. This regulates their diffusion through the extracellular matrix (ECM) and serves as a cofactor, which regulates specificity and affinity for signaling FGFRs [123]. Deregulation and excessive FGF signaling might result in various manner cancer initiation or progression, and the amplification of a number of FGF family members or FGFRs has been described [124,125,126]. Among the several FGF family members that were described, FGF-1 (acidic FGF) and FGF-2 (basic FGF) represent two of the main angiogenesis-related factors [16,127,128], whereas other members have well-defined roles; e.g., in the development of the central nervous system [129]. Both FGFs (1 and 2) were identified in the search for a secreted tumor angiogenic factor postulated by Judah Folkman. In contrast to other family members, these two prototypic angiogenic FGFs lack conventional secretory signal peptides and are apparently exported from producing cells by direct translocation across the cell membrane [123,130]. FGFs are pleiotropic factors acting both in an autocrine and paracrine manner on tumor and stromal cells. Both FGFs were shown to potently stimulate EC proliferation and migration by triggering various effects, including the regulation of matrix metalloproteinase expression [126,131,132]. Apparently, the mitogenic activity of FGF-2 is significantly more powerful than that of VEGF-A [88]. FGF-2 may also be chiefly involved in the evasive resistance to VEGF-inhibition [21,23,126]. Thus, the compensatory upregulation of FGF-2—besides its own potent, perhaps VEGF-independent role in pathological neovascularization—made this signaling pathway a novel target for drug development aimed at simultaneously interfering with both VEGF and FGF-signaling cascades. Brivanib, a dual FGF/VEGF–RTK investigational inhibitor, targeting VEGFR-2 and -3, and FGFR-1, -2 and -3 signaling was experimentally shown in pre-clinical experiments to be efficacious in limiting evasive revascularization and has been clinically tested in hepatocellular (HCC) carcinoma as a second line therapy following multikinase-inhibitor sorafenib failure as well as in patients with various malignancies [133,134,135]. However, brivanib application results only in marginal clinical benefits. Moreover, the first-line therapy in patients with unresectable, advanced HCC showed more side effects than the multikinase inhibitor sorafenib, necessitating the discontinuation of treatment [135]. Recently, other strategies have been developed for the simultaneous inhibition of both signaling cascades by using multivalent decoy receptors (VEGF/FGF-Trap, VF-Trap) (Figure 3) capable of VEGF and FGF-2 quenching. The pre-clinical efficacy testing of the substance in ocular neovascularization settings shows more potent anti-angiogenic potential than anti-VEGF monotherapy [136].

#### 3.2.2. PDGF Family and PDGF Receptors

The PDGF-family is composed of five members containing four (A-, B-, C-, and D) homodimer-building disulfide-bonded polypeptides and a heterodimer built up of AB polypeptide chains. PDGF receptors, known as PDGFRα and -β, belong to the family of type III tyrosine kinase receptors together with stem cell factor (SCF) receptor (Kit), colony-stimulating factor-1 (CSF-1) receptor and Flt-3 [137]. PDGF receptor activation has been observed in neoplasias and other diseases involving excessive cell proliferation, such as fibrosis [137]. PDGF-BB homodimers have been shown to modulate EC proliferation and angiogenesis acting through PDGFRβ. PDGF-B, secreted by ECs, is required for the proper pericytic investment of the endothelial tube, which is achieved by a retention motif to heparan sulfate proteoglycan (HSPG) in the framework of normal angiogenesis. However, this seems to be dispensable in the context of tumor vessels [138,139]. Moreover, pericytes require PDGFRβ for their recruitment to tumor vessels [138,140]. PDGF, secreted by tumor cells, influences both the vasculature and stromal compartment and contributes to the increased intratumoral pressure [141]. The upregulation of PDGF-B and increased pericytic coverage render tumor vessels inaccessible to anti-VEGF agents [142]. Conversely, immature blood vessels that have not yet recruited periendothelial cells are selectively vulnerable to VEGF deprivation [143,144]. The immunological stripping of pericytes might render vessels better accessible for anti-VEGF treatment. The combined inhibition of VEGF and PDGF signaling enforces tumor vessel regression by interfering with pericyte-mediated EC survival mechanisms [145]. However, precautions must be taken given that pericytic coverage plays an important role in limiting tumor cell metastasis [146]. An epithelial-to-mesenchymal transition (EMT) by which transformed cells acquire PDGFR expression, and hence PDGF responsiveness, might correlate with an increased invasiveness of tumors including glioblastoma [137]. Furthermore, PDGF might contribute to the VEGF-resistance of glioblastoma by inducing endothelial-to-mesenchymal transition (EndMT) with a downregulation of VEGFR-2 expression. This can be significantly counteracted by the dual inhibition of VEGFR and PDGFRs, as recently revealed in a pre-clinical glioblastoma model [147]. These findings indicate that the simultaneous inhibition of relevant alternative signaling pathways might open up novel avenues in targeting neoangiogenesis in solid tumors showing resistance to anti-VEGF monotherapies.

#### 3.2.3. Angiopoietin and TIE2

Two members of the angiopoietin family, Ang-1 and Ang-2, are proteins that bind to Tie-2 receptor, a single-pass transmembrane molecule that is preferentially expressed on the vascular endothelium [13,148]. The Ang/Tie signaling pathway contains a further, less characterized, tyrosine kinase receptor (Tie-1) and two additional ligands, Ang-3 and Ang-4, with only the latter being present in humans [149]. Ang-1 is a ligand of Tie-2 tyrosine kinase receptor and has been shown to promote vessel stabilization and maturation via Tie-2 receptor phosphorylation, while it is also endowed with anti-inflammatory and anti-permeability properties [13,150,151]. Ang-2 is an alternative regulator of pathological angiogenesis, playing a crucial role in the tumor “angiogenic switch” and triggering ocular neovascularization via Tie-2 receptor and integrin signaling [69,152]. Ang-2 is induced by hypoxia and is known to destabilize interactions between ECs and perivascular cells leading to pericyte dropout, a phenomenon which is important in the initiation of neovascularization [69,152,153,154]. Ang-2, while promoting the activity of protein tyrosine-phosphatase β (PTPB), mediates the dephosphorylation of Tie-2. This prevents the activation of Tie-2 by Ang-1 [155]. Abnormal Ang-Tie-2 signaling is responsible for altered mural cell and endothelial interactions, which chiefly participate in pericyte/EC dissociation, as revealed in the diabetic retina—one of the most prevalent conditions with pericyte loss [69,156]. Strategies aimed at normalizing Ang-2 levels might thus promote vessel stabilization and the normalization of the perfusion as well as revascularization [44,157,158]. Improved pericytic coverage might protect novel vessel sprouts from pruning [146,159]. Increased expression of Ang-2 at the expense of Ang-1 correlates with poor prognosis in tumors. It is worth noting that Ang-2 plays a role in attracting Tie-2-expressing monocytes/macrophages (TEMs) with pro-angiogenic function [153,160]. Altogether, Ang-2 is an attractive target for combination therapies of tumor angiogenesis [148,161]. Recently, a novel strategy aimed at simultaneous Tie-2 activation and Ang-2 inhibition with ABTAA (Ang2-binding and Tie-2-activating antibody) has been shown to result in the normalization of tumor vasculature, decreased hypoxia, acidosis, tumor growth and metastasis, with a favorable tumor microenvironment also enabling the enhanced delivery of chemotherapeutic agents into tumors [162]. Ang-2 might act synergistically with VEGF in modulating endothelial vascular permeability [163], and might play a role in adaptive tumor resistance to VEGF blockade [164]. The inhibition of Ang-2 has been shown to reduce the anti-VEGF doses required to achieve equivalent therapeutic effects, indicating that these molecules potentiate each other’s activity [165,166]. Pre-clinical studies and clinical trials using combinations of VEGF-A blocking and Ang-2 inhibitors (peptide-Fc fusion “peptibody”), as well as bispecific CrossMab anti-VEGF/anti-Ang-2 Ab (RG7716) (Figure 3), are presently ongoing to estimate their efficacy in tumor and ocular neovascularization [147,148,165,167]. The dual inhibition of VEGF/Ang-2 may delay tumor growth and prolong survival in mice with glioblastoma multiforme (GBM) by normalizing tumor vasculature and reprogramming the tumor immune microenvironment via an effect on the phenotypic polarization of tumor macrophages [168,169].

#### 3.2.4. HGF and c-MET

Hepatocyte growth factor (HGF), also known as scatter factor (SF), and its receptor tyrosine kinase, c-MET (mesenchymal-epithelial transition proto-oncogene), are involved in cell proliferation, motility, migration and invasion. A “cell scattering phenotype” appears also under physiological conditions, not only in the context of various malignancies, as it was observed for hepatocytes and placental invasive trophoblast cells [170]. HGF is a mediator of tumor and stromal interactions via the c-MET receptor and is often found to be overexpressed in the stroma of primary tumors [171]. The transcriptional upregulation and activation of c-MET, without gene amplification, was observed in a number of human tumors, and its regulation by hypoxia has been described [170]. HGF stimulates EC motility and growth [172], and a cross-talk between the VEGF-A and HGF signaling pathways in ECs has been observed, which may promote VEGF-A-driven angiogenesis by enhancing intracellular signaling [173]. Moreover, MET signaling can promote angiogenesis through the induction of VEGF-A expression [174]. Altogether, HGF acts as a cytokine in blood vessel formation via the c-MET signaling pathway. c-MET might play a role in anti-angiogenic therapy resistance in response to VEGF inhibition by promoting an EMT-like phenotype and invasiveness in GBM patients receiving bevacizumab [175]. The inhibition of MET in GBM mouse models blocks mesenchymal transition and invasion provoked by VEGF ablation [175]. MET is a critical player and therapeutic target in tumorigenesis [176,177]; nevertheless, a combination of onartuzumab, an antibody blocking c-MET (Figure 3), with bevacizmab resulted in no additional benefit over anti-VEGF-A monotherapy in recurrent GBM [178].

## 4. Microenvironmental Confounder Factors in Anti-Angiogenesis

Multifactorality and the complexity of interactions in abnormal angiogenesis might limit the success of therapeutic approaches targeting only single molecules. Additionally, special metabolic properties of tumor cells and the significant influence of the stromal microenviroment might often represent unforeseen major obstacles of tumor therapies. An intricate interplay between tumor cells, stroma and tumors infiltrating blood-born inflammatory cells often elicits unfavorable changes of the microenvironment. These alterations combined with tissue-specific/context-dependent mechanisms of neovascularization not only significantly limit drug delivery, but might also modify outcomes of anti-angiogenic treatments. A selection of important confounding factors will be discussed below.

### 4.1. Abnormal Vessel Structure and Differential Sensitivity

Sustained angiogenesis is considered a hallmark of solid tumors [5]. Tumor vessels are heterogeneous and become abnormal in structure and function, compromising perfusion and oxygen delivery [20,179]. Angiogenic responses elicited under experimental conditions upon the overexpression of VEGF164 (the murine cognate of human VEGF165) lead to the identification of various aberrant vessel types, with only two types retaining VEGF responsiveness and hyperpermeable phenotypes. These vessels are the so-called mother vessels (MV) with loosened perivascular coverage derived from venules and the early glomeruloid microvascular proliferations (GMP), respectively. MVs, which give rise to GMPs, develop from preexisting microvessels after pericyte detachment and basement membrane degradation. All other vessel types appear to downregulate the expression of VEGFR-2, rendering them insensitive to anti-VEGF application, which provides an explanation for the differential sensitivity of early and late generated vessels [180]. It is worthy of note that the inhibition of tumor angiogenesis, for instance, would paradoxically lead to the selective survival of hypoxic cancer cells, especially in the center of the tumor mass. This leads not only to the further expansion of the non-perfused tumor mass, but also severely limits the chance of drug delivery to the hypoxic tumor center via the obliterated microvaculature. Patients whose tumor perfusion or oxygenation increases may survive longer. Strategies aimed at alleviating tumor hypoxia as a function of better perfusion could improve the outcomes of tumor therapeutic approaches [158,181]. To achieve the normalization of tumor microvessels is, however, a demanding task. Apparently, an initial transient normalization of microvessels can only be achieved within a plasticity window for microvascular remodeling, which might allow for the optimal targeting of different components of the vessel wall (EC, PC, basement membrane) and inflammatory cells. In order to restore the balance between angiogenic activators and inhibitors without heavy pruning of tumor vessels, a carefully selected dosage of anti-angiogenic (VEGF-targeted) therapies might be of crucial relevance. Although multiple strategies can be deployed to achieve a more regular array of vessels [158,181], recent experimental approaches focusing either on low (metronomic) dosages of VEGF inhibitors or targeting alternative pathways have delivered experimental evidence that vessel normalization not only results in a homogeneous distribution of functional tumor vessels but is accompanied by favorable changes in the tumor microenvironment. Thus, the disruption of the vicious circle fueled by non-productive angiogenesis has an immense benefit for drug delivery [162,182]. The major advantage of this vessel normalization strategy is that it is achieved by reprograming the immunosuppressive tumor microenvironment with a shift towards the immune-stimulatory, proinflammatory M1-like polarization state of tumor-associated macrophages with signs of a favorable effect also on T-cell infiltration [162,182]. Vessel normalization might find an application where the success of conventional anti-angiogenic treatment is limited (e.g., breast cancer, glioma). The lack of validated prognostic and predictive biomarkers, however, represents one of the greatest obstacles in determining treatment outcomes and optimal responses. A recent study provides a possible mechanistic insight into the heterogeneous treatment responses connected to abnormal vessel structure observed in high-grade glioma patients. Thus, the expression of Sox7 is crucial in promoting the abnormal structure of high-grade glioma vessels and is responsible for poor therapeutic outcomes of VEGFR-2 inhibition [183]. Validating the clinical efficacy of vessel normalization is demanding. Histological and morphometric analyses of biopsies in clinical settings are less feasible than in preclinical studies. Nevertheless, non-invasive radiological imaging methods aimed at assessing changes in tumor vascular architecture, integrity, perfusion and metabolism might deliver answers as to the relationship between vascularization parameters, vascular normalization and the normalization window in clinical contexts [184].

### 4.2. Metabolic Switch and Extracellular Acidosis

#### 4.2.1. Metabolic Reprogramming

##### Tumor Cells

Maladaptive responses of both tumor cells and the stromal microenvironment, especially that of immune cells, might fuel angiogenesis and tumor growth [31,185,186]. Hypoxia is not only one of the most obvious stimuli of enhanced pro-angiogenic factor expression [185] but also leads to metabolic reprogramming as an adaptive escape in response to a hypoxic tumor microenvironment [187,188]. A fundamental phenomenon of malignant metabolic reprogramming is known as the “Warburg effect” [187,188,189,190,191,192], whereby tumor cells enter into a permanent glycolytic metabolic pathway as an adaptation to low oxygen tension [188,190]. A wide range of human tumors uses aerobic glycolysis as the main metabolic pathway for generating ATP (adenosine triphosphate) even in the presence of oxygen. A dysregulation of HIF-1alpha coupled with the abnormal expression of metabolic enzymes (pyruvate dehydrogenase complex) during cancer development might play a role in inducing the Warburg effect [53,193]. The deviation from the default oxidative phosphorylation (OXPHOS) program is most likely to be further supported by the generation of a reversed pH gradient in cancer cells (high pH inside/low pH outside) as a consequence of increased proton transporter expression Na+/H+ exchanger isoform 1 (NHE1). Tumor cells extruding protons (H+) in this way raise their intracellular pH, and a shift towards intracellular alkalosis blocks Krebs-cycle (TCA) and OXPHOS is achieved [47,194]. Recent observations of aggressive triple-negative breast cancer (TNBC) cells, however, confirmed a hybrid metabolic phenotype as part of a metabolic plasticity cancer which cells might deploy to switch their metabolism phenotypes between glycolysis and OXPHOS during tumorigenesis and metastasis. This indicates that targeting both glycolysis and OXPHOS might be necessary to eliminate their metabolic plasticity [195].

##### Cancer-Associated Fibroblasts

Cancer/tumor-associated fibroblasts (CAFs/TAFs) might also contribute to important metabolic changes by turning into a factory for the production of energy-rich metabolites by aerobic glycolysis, covering the increased energy demand necessary for extensive tumor cell proliferation [196]. This phenomenon is described as the “reverse Warburg effect“. The increased dependence of cancer cells on the glycolytic pathway for ATP generation provides a biochemical basis for the design of therapeutic strategies to preferentially kill cancer cells by the pharmacological inhibition of glycolysis [187,197]. The metabolic targeting of HIF-dependent glycolysis was shown to reduce lactate, increase oxygen consumption and enhance the response to high-dose single-fraction radiotherapy in hypoxic solid tumors in a preclinical model setting [198].

The role of CAFs/TAFs in modulating the tumor microenvironment and influencing the behavior of neoplastic cells is not exhausted by the above glycolytic changes [72,199]. Indeed, while producing ECM, which can mechanically compromise microcirculation and drug delivery, CAFs secrete a plethora of cytokines including VEGF-A. These factors promote angiogenesis and tumor cell proliferation and play an aberrant stimulatory role during chronic inflammatory states by recruiting immunosuppressive cell populations in cancer; additionally, they contribute to the refractoriness upon anti-angiogenic treatment by the compensatory production of pro-angiogenic factors other than VEGF-A [24,34,72,199,200,201,202]. CAFs contribute to the evasion of immune surveillance of tumor cells and induce angiogenesis and tumor growth [72,199,200]. Altogether, modulating CAF function might have important therapeutic implications and could have a number of facets. Thus, besides pharmacological tools interfering with glycolytic processes, targeting the stromal barrier, CAF-secreted factors, and ECM interactions; blocking CAF activity; and transforming CAFs or eliminating CAFs by T-cell mediated mechanisms might all belong to the repertoire of successful anti-cancer practices [72].

##### ECs

ECs similar to tumor cells were shown to rely on glycolysis for ATP production with a glycolytic rate similar to tumor cells that is further increased as soon as ECs become activated in conjunction with sprouting angiogenesis in preclinical neovascularization models. The glycolytic flux in migratory tip cells is particularly high [203]. Targeting EC metabolism in addition to growth factor blockade is emerging as a novel avenue for manipulating the microvasculature [22]. This makes 6-phosphofructo-2-kinase/fructose-2,6-bisphosphatase 3 (PFKFB3)—a key regulator that controls the balance of tip versus stalk cells in sprouting angiogenesis—a potential druggable target of the glycolytic pathway. Results of a pharmacological blockade of PFKFB3 with 3-(3-pyridinyl)-1-(4-pyridinyl)-2-propen-1-one (3PO) showed a partial transient reduction of glycolysis leading to attenuation of pathological angiogenesis in vivo in pre-clinical angiogenesis models [203].

#### 4.2.2. Extracellular Acidosis

The host microenvironment is essential in regulating tumor cell behavior and angiogenic stimuli [48]. Extracellular acidosis in solid tumors (Figure 1) is an end result of high glycolytic flux (increased metabolism of glucose) and poor vascular perfusion [204,205]. The low extracellular pH has further consequences. In addition to the hypoxia-driven stimulation of VEGF-release, the production of this angiogenic factor is also stimulated by low pH, indicating that an acidic tumor microenvironment contributes to tumor angiogenesis and progression [206,207]. As a low pH under oxygenated conditions stimulates VEGF expression, hypoxia and acidosis could independently up-regulate VEGF [208]. A tumor-derived lactic acid-induced expression of VEGF by an HIF1α-driven mechanism has recently been shown in tumor-associated macrophages [209]. Furthermore, a low pH was shown to increase the release of active cathepsin B—an important matrix-remodeling protease that might be important in the angiogenic switch [210,211].

Altogether, the extracellular acidic tumor microenvironment supports cancer cell growth and migration and blunts the response of the immune system. Its contribution to microenvironmental resistance by either affecting drug permeability or altering the physicochemical properties of the chemotherapeutic agents has been described [194]. Thus, in addition to xenobiotic transporter-mediated resistance—e.g., MDR1 (multidrug resistance protein 1; also known as P-glycoprotein) expression—tumors have evolved a mechanism that allows them to take advantage of an unfavorable oxygen supply by reprogramming their metabolism and developing non-specific physico-chemical barrier mechanisms leading to an “ion trapping” phenomenon, which has a negative impact on weak base delivery [205,212]. Apparently, hypoxic conditions are frequently associated with cellular resistance to conventional anticancer drugs [187]. The uptake of charged drugs by tumors is greatly compromised. In particular, those with a weak base character are negatively affected by this unusual pH gradient [204,205]. Tumors of the bladder, kidney and gastrointestinal system in particular are exposed to extremes of pH values [204].

### 4.3. Tumor Microenvironment and Deregulated Inflammatory Responses

Inflammation is a critical component of tumor progression and is a distinguishing feature of pathological neovascularization over physiological angiogenesis [31,49,76]. The tumor-driven recruitment of inflammatory cells contributes significantly to the “angiogenic switch” [31,49,50], and the degree of inflammatory infiltration often inversely correlates with prognosis in solid tumors [213]. Neutrophils and monocytes contribute in a time-shifted way to inflammation, whereas mast cells contribute both in early and late phases, as described for wound-healing [76]. Tumor-associated macrophages (TAMs) (Figure 1) are particularly abundant and are present at all stages of tumor progression. They can stimulate angiogenesis, enhance tumor cell invasion and suppress cytotoxic anti-tumor T-cell responses through the expression of an array of effector molecules [213]. Recruited cells, besides expressing pro-angiogenic growth factors, regulate the bioavailability of VEGF by producing proteases (e.g., MMP9) [55,76]. Thus, both direct and indirect modulatory roles are assigned to these cells in tumor neoangiogenesis. Governed by hypoxia and the cytokine microenvironment of the stroma, infiltrating inflammatory macrophage cells might display either immunostimulatory M1 or immunosuppressive/pro-angiogenic M2 phenotypes [49,214,215]. The acidic tumor microenviroment represents a direct trigger for M2-like polarization and the VEGF-expression of TAMs via HIF1, which contributes to the maintenance of immunosuppression [209]. Whereas the contribution of TAMs is extensively investigated in the context of the tumor microenvironment, these macrophages are not unique among the attracted inflammatory cells known to potentiate neoplastic processes [76]. Thus, the role of other blood-born myeloid cell types, and the contribution of cells of the adaptive immune system, has become more established in terms of modulating the tumor microenvironment, with a significant (negative) impact on antitumor immunity and angiogenic escape [49,50,216]. Thus, a special hematopoietic lineage of proangiogenic monocytes, the TEMs (see also Section 3.2.3), was shown to produce VEGF and WNT7b (wingless-related integration site 7b), which could significantly contribute to the stimulation of neoangiogenesis and hence to tumor growth [6,213,217]. The selective depletion of TEMs in pre-clinical models inhibits tumor angiogenesis and growth [217]. In addition, the contribution of the adaptive immune system is becoming more acknowledged in tumorigenesis [218]. Regulatory T-cells (Treg) have been shown to crucially limit the antitumor immune response known to compromise the success of tumor immunotherapies by promoting angiogenesis as well as tumor growth. 

Hypoxia owing to compromised tissue perfusion is a significant but largely neglected confounding factor to the therapeutic efficacy of anti-tumor treatments [219]. Hypoxia disturbs both innate and adaptive immune reactions, with profound effects on cancer treatment outcomes [209,220]. Anti-tumor functions of immune cells are downregulated largely in response to tumor-derived signals [34]. Hypoxia was found to promote immunosuppressive processes by shifting the polarization of macrophages towards an M2 phenotype and increasing Treg activity while suppressing CD4+ effector T cell (Teff) function [34,219,220,221,222]. Stromal fibroblasts (CAFs) synergize with immunosuppressive alternatively activated M2-polarized macrophages during tumor progression [223]. The success of anti-cancer approaches on mid- and long-term scales is pivotally governed by innate and adaptive immunity [216]. Therapeutic interventions might support the “re-education“ of TAMs or other infiltrating cells, changing them into being immunostimulatory [182,224]. Reducing/modulating hypoxia by various approaches could significantly contribute to improved therapeutic responses by reprogramming the immunosuppressive tumor microenvironment [162,182,220]. For instance, the normalization of the abnormally leaky, tortuous microvasculature and reducing the overt turgor of tumors by promoting degradation or modifying the deposition of excessive ECM could significantly support tumor tissue reperfusion [162,182,219,225]. Moreover, approaches that modulate Treg homing or activity might represent important future therapeutic targets in the context of tumor angiogenesis [218].

Besides hypoxia, somewhat paradoxically, therapy-induced cancer cell death could act as a further confounding factor as it significantly promotes the growth of surviving neoplastic cells by a macrophage-dependent dead cell-mediated tumor-promoting inflammation. Recent advances in signaling mechanisms revealed that this process is modulated by a class of lipid mediators, resolvins, that promote the uptake of cancer cell debris, thus restricting cancer-promoting inflammation and cancer growth [226].

## 5. Therapeutic Modalities

Since the identification of VEGF-A as a major pro-angiogenic factor, strategic efforts have often followed a single-target approach with the aim of down-modulating the VEGF-A/VEGFR2 signaling axis in fighting neoangiogenesis [10,15]. Since the discovery and FDA regulatory approval of the first pan-VEGF-A inhibitor, the monoclonal antibody bevacizumab, a number of disparate molecular tools have been developed aimed at inhibiting tumor angiogenesis. In general, treatments with anti-angiogenic agents (Table 1) use one of the two major principles to interfere with angiogenic/VEGF signaling: (a) restricting the ligand-mediated activation of a particular receptor either by modulating the bioavailability of the cognate ligand (i.e., a pro-angiogenic molecule) or interfering with the accessibility of the ligand-binding site of the receptor for this pro-angiogenic ligand (Figure 2 and Figure 3); and (b) inhibiting down-stream signal cascade activation even in the presence of receptor occupancy by blocking the kinase activity of RTKs (Figure 2 and Figure 3) [10,227,228]. Although the blockage of the VEGF/VEGFR-axis might be a plausible target of anti-angiogenic therapies for a number of tumor types, responsiveness to a given form of anti-angiogenic therapy could, however, be significantly influenced by stage and tumor-type specificities of neoangiogenesis. The heterogeneity of angiogenic responses was also observed, even in normal tissues that were experimentally challenged by VEGF-application. Thus, VEGF elicits a more intense and longer-lasting response in certain organs than in others [77]. Efficient angiogenesis inhibition due to the heterogeneity of ECs and/or tumor vessels is almost certainly precluded with a single molecule/agent approach in the context of solid tumors. Moreover, VEGF dependence, maturation state, pericytic coverage, and the stromal (cytokine) microenvironment are all pivotal factors that crucially influence the accessibility and thus the success of the anti-angiogenic targeting of the heterogeneous vessel population embedded in the tumor stroma [45,225,229]. Thus, pancreatic carcinoma remains often treatment-refractory to anti-VEGF agents, and treatment of hepatocellular carcinoma with anti-VEGF tools, in spite of significant hypervascularization, is of limited suitability [10,230]. The recognition of alternative molecular pathways of angiogenesis causal to intrinsic resistance or compensatory (acquired) evasive resistance in response to anti-VEGF therapies initiated the development of tools targeting VEGF-A-independent molecular pathways (Figure 3) [13,33,231,232]. However, the currently approved therapeutic approaches are still much too VEGF-centered or appear to have little target specificity, as is the case with small molecule tyrosine-kinase inhibitors.

### 5.1. Large Molecules

#### 5.1.1. Biologics Targeting VEGF Ligands

##### Bevacizumab

The first VEGF-A inhibitor that received regulatory approval by the US Food and Drug Administration (FDA) was bevacizumab, in 2004, for the first-line treatment of metastatic colorectal cancer [15]. Bevacizumab (Avastin) is a humanized pan-anti-VEGF-A mAb derived from a mouse monoclonal clone “A.4.6.1.“ which specifically recognizes and neutralizes all bioactive isoforms of human VEGF-A [15,234]. By quenching isoforms of VEGF-A (Table 1; Figure 2), it prevents the activation of VEGFR-1 and -2 and inhibits tumor growth, as revealed in human xenograft models [235]. Bevacizumab received regulatory approval in the US (FDA) and Europe (EMA) as a first and second-line treatment of metastatic colorectal cancer and as a first-line treatment for metastatic non-small-cell lung cancer and metastatic renal cell carcinoma. Furthermore, it is approved as a first-line treatment in recurrent glioblastoma in the US and in metastatic breast cancer and metastatic ovarial cancer in Europe [15,236]. However, the regulatory approval of bevacizumab for treating metastatic breast cancer has been withdrawn by the FDA in the US (2011). Furthermore, bevacizumab has been approved as a combination therapy for the first and second-line treatment of metastatic colorectal cancer and metastatic renal cell carcinoma [15,55]. Adverse effects associated with anti-VEGF Avastin therapy were reported as gastrointestinal perforation, thromboembolic events, pulmonary embolism, hypertension, gastrointestinal hemorrhage, and cerebral hemorrhage or vascular accident [237].

##### VEGF-Trap

VEGF-Trap (Aflibercept, AFL) is a highly potent decoy receptor for all VEGF-A, VEGF-B and PlGF isoforms (Table 1; Figure 2). It was developed as a therapeutic alternative to circumvent evasive resistance to anti-VEGF-A treatments [231,238,239,240]. AFL is a soluble recombinant chimeric protein formed by the combination of the immunoglobulin G constant region with the VEGF-binding domains of VEGFR-1 and VEGFR-2 (extracellular Ig-like domain number 2 of VEGFR-1 and the extracellular Ig-like domain number 3 of VEGFR-2) [231]. Compared to other anti-VEGF-A agents, AFL binds VEGF-A with higher affinity and could offer a more prolonged inhibitory effect [241,242]. AFL complexes with tissue or tumor-derived VEGF are not cleared from systemic circulation as rapidly as antibody complexes [243]. Recent studies indicate that AFL forms a monomeric homogenous 1:1 complex with dimeric VEGF-A, in contrast to bevacizumab [244]. Moreover, AFL also occludes the heparin-binding site on VEGF165 besides blocking the amino acids necessary for VEGFR1/R2 binding [244]. Large multimeric bevacizumab VEGF-A complexes can lead to platelet activation and binding to endothelial cells. Moreover, treatment with bevacizumab has been associated with arterial thromboembolism in colorectal cancer patients [244,245]. The lack of multimeric immune complex formation and the induction of thrombocyte activation might be expected to cause fewer undesirable effects of AFL than bevacizumab, even by systemic administration, at least in animal models [243,245]. Moreover, substances targeting only VEGF-A expected to exert no overt effect on the inflammatory activation of microglial/macrophage cells. This is partly due to the lack of VEGFR2 on inflammatory cells and the possible compensatory upregulation of PlGF upon VEGF-inhibition [246,247]. Previously, PlGF deletion or inhibition and VEGFR-1 ablation were shown to affect macrophage polarization coupled with vessel normalization in tumors [248], while no significant decrease in the number of infiltrating cells appeared [249]. Besides being a decoy receptor for the ligands of VEGFR-1 and VEGFR-2, AFL has been shown to bind Galectin-1 [250]. Galectin-1 has been reported to underlie the limited success of anti-VEGF agents. Galectin-1 is a secreted carbohydrate-binding lectin that activates down-stream targets of VEGFR2 even in the absence of canonical VEGF ligands and thereby plays a role in the evasive resistance to anti-VEGF-A treatment [251,252]. Between 2011 and 2016, AFL gained regulatory approval for the treatment of ocular neovascular diseases and as a second-line systemic treatment for metastatic colorectal cancer [15,253].

#### 5.1.2. Biologics Targeting VEGFR2

##### Ramucirumab

A fully-human IgG1 mAb that binds to the ligand-binding site of VEGFR-2 preventing the activation of this receptor (Table 1; Figure 2) has been developed (ramucirumab, IMC-1121b) [254] and has received approval for use as a monotherapy or in combination with chemotherapy in metastatic gastric/gastroesophageal (GE)-junction adenocarcinoma, and in combination with chemotherapy for metastatic non-small cell lung cancer and for metastatic colorectal cancer (mCRC) [255]. The activation of VEGFR-2 by proteolytically processed, mature VEGF-C and -D gives a rationale for the use of this substance [256,257] (see also Section 3.1.). Ramucirumab is able to inhibit the binding of VEGF, VEGF-C and VEGF-D to a soluble extracellular domain of human VEGFR-2 [255]. Several clinical trials are underway estimating the clinical efficacy of ramucirumab [258]. One of the potential pitfalls of applying anti-VEGF-receptor antibodies, however, is a potential acute increase of circulating VEGF levels due to the displacement of VEGF from its receptors [259].

### 5.2. Small-Molecule Multikinase Inhibitors

In addition to proteinaceous biological tools targeting VEGFR2 or the bioavailability of its ligands, strategies for inhibiting the activity of angiogenic receptor tyrosine kinases (RTKIs) at the catalytic domain in a competitive or allosteric way have evolved as systemic treatment modalities for cancer (Table 1; Figure 2 and Figure 3) [17,228,260]. These molecules, even if the primary intention is often the down-modulation of the VEGF/VEGFR2 axis, have broader affinities to multiple receptor tyrosine kinases and are therefore considered as multikinase inhibitors [236]. The first kinase inhibitors which gained regulatory approval were for the treatment of advanced RCC patients with deregulated pVHL expression [15,227,236,260]. Later on, the approval of such substances was extended to treatment for other conditions, such as HCC [229]. In spite of their beneficial anti-tumor activity in certain malignancies, clinical resistance and toxicities limit the efficacy of these drugs [15,18,230]. Moreover, long-term blockade with small-molecule kinase inhibitors has proven more difficult to achieve than with biological tools [18]. The first agents approved for advanced RCC were sorafenib, sunitinib, and pazopanib, and these were reported to display adverse effects unrelated to VEGFR-blockade. This is very likely attributed to the fact that they also bind other RTKs, including PDGFRs, c-kit, Flt3, RET, CSF1R, and B-Raf. Some inhibitors can also block FGFR1 (sorafenib) and EGFR (vandetanib) or even the non-RTK B-Raf (sorafenib) [227,261]. Sorafenib has both anti-proliferative and anti-angiogenic effects on tumors. Sorafenib was initially isolated as a Raf inhibitor, and it was discovered to act as an allosteric inhibitor of VEGFR and PDGFR tyrosine kinase signaling. It is the first TKI drug approved for the treatment of advanced HCC [230] and thyroid cancer [15]. TKIs of the second generation, tivozanib and axitinib, are endowed with more selectivity to VEGFRs and have an improved potency. This holds especially true for axitinib [236]; this substance is also approved for the second-line treatment of metastatic RCC refractory to sunitinib [15,236]. The further indication of some of these substances includes pancreatic neuroendocrine tumors [262]. While none of the above substances were approved for mCRC, Regorafenib, an oral inhibitor of angiogenic, stromal and oncogenic TKs [263,264], was recently approved as a second-line monotherapy treatment in mCRC and also for HCC (EMA/451550/2017). Pre-clinical testing of Regorafenib in a combination setting also reveals promising results [263]. However, combination therapies of RTKs with cytotoxic substances or with bevacizumab resulted in no further benefits and eventually had worse side effects than in monotherapy [15].

## 6. Future Perspectives—Possible Answers to Therapy Resistance

### 6.1. Metronomic Therapy Regimes

Metronomic chemotherapy, based on the continuous administration of low-dose cytotoxic agents without extended intervals, is an emerging potential method of reducing side effects of high-dose bolus treatments in various settings for cancer treatment [265,266]. More interestingly, several cytotoxic drugs have anti-angiogenic properties if administered frequently and at lower doses compared with standard schedules containing maximal tolerated doses (MTD). In this way, instead of a direct cytotoxic effect on malignantly transformed cells, cytototoxic substances most likely target the tumor microenvironment and impair tumor ECs resulting in the restriction of angiogenesis. Thus, slowly proliferating tumors might be kept in a dormant, non-metastatic state. Although the exact mechanisms are not known, a reduction of VEGF expression has been observed [266,267,268]. In addition, metronomic regimes might also be efficient against advanced metastatic disease when combined with explicit angiogenesis inhibitors; e.g., bevacizumab [269,270,271,272].

### 6.2. Drug Repurposing

The repurposing or indication switching of already approved drugs might represent a novel focus of anticancer/anti-angiogenic therapies, saving significant time, resources and yielding fewer unfavorable side effects [273,274,275]. A wide variety of antipsychotic drugs are now reported to have potent anti-cancer properties against a number of malignancies, in addition to their antipsychotic effects [274]. One of the early examples of such a potential indication-switching trial is the generic, globally-used anticonvulsant, Valproate (VPA)—a short-branched fatty acid molecule. VPA appears to be a histon-deacetylase (HDAC) inhibitor. HDACs are considered as potential anticancer drugs with multimodal properties [276] including potent angiogenesis inhibitor activity which alters VEGF signaling [277]. In line with this, HDACs were found to induce angiogenesis by the negative regulation of tumor suppressor genes, and HDAC inhibition significantly downregulates HIF1α and VEGF expression [278]. However, HDAC inhibitors might exert pleitropic effects that are not exclusively attributed to epigenetic chromatin modifications as protein acetylation, similar to phosphorylation, might serve as an additional post-translational regulatory mechanism of non-histone and cytoplasmic proteins [279,280,281]. Recently, the regulation of VEGFR-2 activity has been shown to be reversible by acetylation [282]. Thus, interfering with this process by means of HDAC inhibitors could represent an alternative target, and current HDAC inhibitors might serve as templates for designing new anti-angiogenic/anti-cancer drugs with more potency and selectivity [283]. Clinical trials are underway to test new HDAC derivates in advanced solid tumors and hematologic malignancies [284]. Moreover, new strategies and clinical trials are emerging using HDAC-inhibitors in combination settings to increase their efficacy in various malignancies [281]. On the other hand, the modulation of T-cell function and epigenetic reprogramming by HDAC inhibitors might be an interesting avenue of manipulating immune–oncological mechanisms [285].

### 6.3. Reprogramming the Immunosuppressive Microenvironment

Due to the significant contribution of the tumor microenvironment to therapy resistance against cytotoxic substances as well as anti-angiogenic treatments, new innovative tools are emerging aimed at targeting the immune microenvironment of tumors by manipulating the activation of CD8+ T-cells. Preventing the premature exhaustion of these cells could have several beneficial effects that indirectly could also have a repercussion on the vascularization of tumors [286,287,288]. Immune checkpoint blockers (ICBs) might represent a paradigm shift in reprogramming the immunosuppressive tumor microenvironment and immune evasion [286]. Immune checkpoint blockers were designed to interfere either with receptors and/or their cognate ligands of the “immune checkpoint pathway” (i.e., intrinsic negative regulators of T-cell function) in order to increase antitumor immunity [288]. T cell-expressed receptor CTLA-4 (the cytotoxic T lymphocyte-associated antigen 4) or PD-1 (programmed cell death receptor) on T cells and its ligand PD-L1 expressed by antigen-presenting cells/tumor cells serve as molecular targets for targeting this pathway. Humanized monoclonal antibody tools directed against PD-1, PD-L1 and CTLA-4 were approved as ICBs: anti-PD-1—pembrolizumab, nivolumab—anti-PD-L1—atezolizumab, avelumab, durvalumab—and anti-CTLA-4—ipililumab [289]. ICBs gained regulatory approval for the treatment of melanoma, non-small-cell lung cancer, renal cell carcinoma, urothelial carcinoma, microsatellite instability high or mismatch repair-deficient solid tumors (e.g., colorectal cancer) [290,291]. Particularly promising is the application of immune check-point inhibitors in treating micro-metastatic melanoma or glioblastoma as an early neoadjuvant therapy. This application has a significant modifying effect on the intratumoral microenvironment and systemic responses resulting in reduced tumor size as revealed in glioblastoma patients with resectable/recurrent malignancies before surgical intervention [292,293]. Despite the success of immunotherapies targeting check-point pathways, significant side effects might be associated with checkpoint blockade, including autoimmune processes [289,291,294,295]. Moreover, the success and long-term benefits of immune check-point blockade are not only dependent on a pre-existing T-cell infiltration (as a prognostic predictor) but also crucially affected by the downregulation of cytotoxic CD8 T-cell activation and subsequent T-cell exhaustion. The identification of responders requires a careful analysis of predictive markers [296]. Whether the manipulation of the immune microenvironment could counteract the angiogenic effects of Tregs [219] or promote the efficacy of anti-angiogenic substances will be the subject of further experimental and systematic clinical studies. Nevertheless, the results of vessel normalization experiments hint at their potential beneficial contribution to an immunostimulatory microenvironment [162,182]. Moreover, the hypoxia-induced direct regulation of PD-L1 via HIF1α on immunosuppressive myeloid cells has been recently demonstrated, and its blockade under hypoxia was shown to abrogate myeloid cell-mediated T cell-suppression by modulating cytokine expression [220]. All these findings provide a rationale for a combination of anti-angiogenic strategies with the direct manipulation of immune checkpoint regulation.

## 7. Conclusions

Approaches aimed at targeting tumor neoangiogenesis may only be successful if the higher order of complexity of the tumor microenvironment is considered. The significant heterogeneity of tumors and tumor vessel growth, as well as the number of accessory cellular players involved, provide rationale for multimodal therapeutic approaches in place of monotherapies. While interfering with colorectal-cancer via manipulating the VEGF pathway appears more feasible than treating ovarian or pancreatic tumors with this approach, a deeper understanding of the mechanisms causing reduced responsiveness to anti-angiogenic treatments is required. In particular, the mechanisms of the complex interplay between tumor and non-tumor (stromal) cell types must be disentangled if therapeutic approaches with the aim of the long-lasting restriction of malignancies are to be achieved. More accentuated and selective targeting of alternative angiogenic factors and molecular mechanisms responsible for generating an immunosuppressive/proangiogenic microenvironment within the tumor stroma may lead to better clinical outcomes in the future [286]. In this process, however, the clear identification of druggable biological targets and predictive biomarkers of treatment responses is of crucial relevance and requires a paradigm change from the current “compound-to-trial“ to a future “biology-to-trial“ approach [297,298].

## Figures and Tables

**Table 1:** Approved angiogenesis inhibitors for treatment of human cancer patients [233].

**I. Biologicals**				
**Antiangiogenic agent**	**Trade name**	**Class**	**Target**	Indication *
*Bevacizumab*	Avastin (Mvasi)	MAb	VEGF-A isoforms	m/rCC, mCRC, rGB, m/rNSCLC, rOEC, rFTC, rPPC, mRCC
*Ziv-Aflibercept*	Cyramza	Recombinant fusion protein	VEGF-A, PlGF, VEGF-B, processed VEGF-C processed VEGF-D	mCRC
*Ramucirumab*	Zaltrap	MAb	VEGFR-2	mCRC, mNSCLC, a/mGAC or GEJAC
**II. Small molecule multi kinase inhibitors (MKIs)**				
*Sunitinib malate*	Sutent	MKI	VEGFRs, PDGFRb, KIT, FLT-3 (CD135), CSF1R, RET	GIST, p/a/mPC, a/rRCC
*Sorafenib tosylate*	Nexavar	MKI	VEGFRs, PDGFRs, KIT, FLT-3 (CD135), CSF1R, RET, Raf	HCC, aRCC,p/r/mTC
*Pazopanib hydrochloride*	Votrient	MKI	VEGFRs, FGFRs, KIT	aRCC, aSTC
*Axitinib*	Inlyta	MKI	VEGFR1-3	aRCC
*Regorafenib*	Stivarga	MKI	VEGFRs, PDGFRs, FGFRs, KIT, TIE2, Raf	mCRC, mGIST, HCC
*Cabozantinib-S-malate*	Cometriq	MKI	VEGFRs, RET, MET, TIE2, FLT-3	HCC, p/mMTC, aRCC
*Vandetanib*	Caprelsa	MKI	VEGFRs, EGFR, RET, TIE2	a/mMTC
**III. Other molecules**				
*Everolimus*	Afinitor	S/ThKI	mTOR	BC, a/mPC, aRC, SEGCA

Abbreviations: a, advanced; m, metastatic; p,progressive; r, recurrent; BC, breast cancer; CC, cervical cancer; CRC, colorectal cancer; FTC, fallopian tube cancer; GAC, gastric (stomach) adenocarcinoma; GB, glioblastoma; GEJAC, gastroesophageal junction adenocarcinoma; GIST, gastrointestinal stromal tumor; HCC, hepatocellular carcinoma; MTC, medullary thyroid cancer; NSCLC, non-small cell lung cancer; OEC, ovarian epithelial cancer; PC, pancreatic cancer; PPC, primary peritoneal cancer; RCC, renal cell carcinoma; TC, thyroid cancer; SEGCA, subependymal giant cell astrocytoma; STC, soft tissue carcinoma; MAb, monoclonal antibody; S/ThKI, serine/threonine kinase; CSFR1, Colony stimulating factor 1 receptor; EGFR, epidermal growth factor receptor; FGFR, fibroblast growth factor receptor; FLT-3, fms like tyrosine kinase 3; KIT, v-kit Hardy–Zuckerman 4 feline sarcoma viral oncogene homolog; mTOR, mammalian target of rapamycin; MET, mesenchymal-epithelial transition proto-oncogene; PDGFR, platelet-derived growth factor receptor; PlGF, placental growth factor; TIE2, tyrosine kinase with immunoglobulin-like and EGF-like domains 2; RET, receptor tyrosine kinase proto-oncogene; VEGF, vascular endothelial growth factor; VEGFR, vascular endothelial growth factor receptor.
